# Circus Use by Occupational Therapists: A Collective Case Study

**DOI:** 10.1155/oti/1220112

**Published:** 2025-03-08

**Authors:** Jill Maglio, Carol A. McKinstry, Tracy L. Fortune

**Affiliations:** ^1^Department of Occupational Therapy, Rural Health School, La Trobe University, Bendigo, Australia; ^2^La Trobe Rural Health School, La Trobe University, Bendigo, Australia; ^3^School of Allied Health, Human Services & Sport La Trobe University, Melbourne, Australia

**Keywords:** community, global health, occupation, social circus

## Abstract

**Background:** Circus use by occupational therapists is an emerging practice area with limited evidence.

**Purpose:** The study is aimed at exploring occupational therapists' current use of circus and identifying the potential for broader applications addressing both individual and community needs.

**Method:** Purposive sampling was adopted to recruit eight circus-using occupational therapists to participate in this collective case study. Semistructured interviews yielded qualitative data, which were coded and thematically analyzed.

**Findings:** Circus is being used primarily to address performance capacity limitations but with an awareness of its potential to address broader community and sociopolitical needs. Analysis yielded two distinct “cases.” The first, *individual-focused circus*, exemplifies how circus is used to address performance capacity, while the second, *community-focused circus*, describes current and envisaged future circus use as community development.

**Conclusion:** There is potential to move toward a more occupation-focused and community-driven use of circus in sociopolitical contexts. Further exploration is needed into the therapeutic benefits of circus use by occupational therapists. The inclusion of educational content that builds students' capacity to adopt community development approaches in practice, alongside enhanced understanding of collaboration benefits between occupational therapists and “activist” disciplines, is paramount, if we are to address occupational injustices and promote occupational rights.

**Summary**

• Circus use by occupational therapists reflects a predominantly individual remediation focus with potential for more community-centered practice.
• Given the limitations of many occupational therapy models, CanMOP [1] may be beneficial for occupational therapists using circus to promote community development.
• Collaboration among occupational therapy, community development, and social circus professions can support more progressive occupational therapy practice, which can address occupational injustice and promote occupational rights.

## 1. Introduction

Circus is an emerging area of practice for occupational therapists despite the profession's long history of using creative arts and crafts as an occupation for health [2]. This study set out to increase understanding of how circus is currently being used globally by occupational therapists and explore possibilities for occupational therapists' use of circus to address more complex societal problems. Circus has a rich history, although its use and meaning have changed through the ages with different types of circus, such as traditional circus, new circus, and community (or social) circus evolving with similar skills and techniques while altering in purpose and values.

A common theme in circus arts literature, more prevalent in historical, drama studies and education contexts, relates to how circus continually evolves to reflect current social trends in a progressive society, thus making it political [3–5]. A perspective exists of circus as an artform that historically valued and celebrated marginalized people for their uniqueness not their conformity [6]. New circus gained momentum in various nations during the 1970s, evolving with an intention to make audiences think and contemplate society as a mechanism in which to invoke activism [3, 5, 7]. Activism is aimed at creating environments that are reflective of the needs of the majority including the occupations people pursue. The choice and autonomy associated with new circus throughout its evolution could be perceived as being complementary to occupational justice. Occupational therapy scholars such as Hammell and Iwama [8], Townsend et al. [9], and Picotin et al. [10] formally espouse the notion of increasing the focus on occupational justice as a direction for practice.

Social or community circus refers to process-focused circus arts programs that are aimed at creating sociopolitical consciousness and capacity-building opportunities for people in diverse living conditions. Social and community circus are differentiated from new circus and traditional circus due to the shift in focus from the production (performance) to the process, where there are no audience members, but everyone is a participant engaging in circus activities. The various activities inherent within circus present an opportunity to therapeutically address performance capacities such as balance, emotional regulation, and hand–eye coordination [11]. Performance capacities are required for participation in many occupations. Engagement in occupations using creative arts can benefit individuals and their relationships with others, including the wider community [12].

Commonly used occupational therapy models guide practice with individuals, and despite most models conceptualizing the environment as central, it is likely that, in practice, a client's broader community and social environment are not in focus. Occupational therapy, practiced in this way, focuses on changing an individual to fit the environment [13]. Community development has been identified as an important domain of practice for occupational therapists to improve not only individual capacities but also community cohesion and community action to challenge inequalities [14]. Social circus has been characterized as accessible and socially transforming through its promotion of autonomy and creative development of participants' critical thinking skills, which may strengthen socioproductive work opportunities [15]. Occupational therapists have been called to work beyond the individual and consider communities as clients by many occupational therapy scholars [8, 14, 16, 17]. Consistent with these calls to action, the Canadian Model of Occupational Participation (CanMOP) focuses on equity and providing social services for people who are marginalized by environmental injustices [1].

Social (community) circus promotes participation in activities that increase life skills acquisition, community integration, and social and emotional health [18, 19]. Community health, as defined by the World Health Organization (WHO), refers to the “environmental, social, and economic resources to sustain emotional and physical well-being among people in ways that advance their aspirations and satisfy their needs in their unique environment.” [20]. Aligned with both a community health paradigm and occupational therapy values, social circus provides creative ways for people to become self-actualized, transforming inequalities that limit life choices and freedom of expression [3, 4]. The occupational therapy profession has similarities with community health paradigms based on its science-driven, globally connected and diverse practices in meeting society's occupational needs [21]. This study is aimed at exploring how occupational therapists are using circus within individual, community, and social–political contexts.

## 2. Methods

The motivation for this study is based on the first author's experience as a circus performer, occupational therapist, and activist. The first author currently uses circus as the therapeutic medium with school-aged children and youth. She also works part of the year in her charity foundation to support people experiencing occupational deprivation, such as refugees and incarcerated persons. The coauthors are both academics interested in emerging areas of practice, particularly exploring the largely untapped potential of occupational therapists working with communities and in health promotion. They joined with the first author as doctoral supervisors to conduct this international study.

Collective case study approaches explore multiple instrumental cases, which are analyzed individually and compared to each other [22]. The collective case study approach was selected to increase understanding of occupational therapists' circus practices in various contexts, informed by different conceptual or theoretical models, with multiple and diverse populations. The analytic strategy involved discovering the meaning within individual pieces of data and then combining those meanings to better understand the phenomenon the research question intended to answer [23, 24]. Understanding the similarities, differences, and trends that exist within occupational therapists' use of circus is paramount before the impacts and benefits can be assessed.

Potential participants were recruited in several ways, including snowballing. Postings on specified social media groups relating to occupational therapy or social circus and listservs related to circus research invited interested occupational therapists to contact the first author.

Purposive sampling was used to intentionally recruit instrumental case participants who could provide experiential knowledge about using circus as occupational therapy. An international perspective was sought to characterize occupational therapists' use of circus in various contexts. Inclusion criteria required participants to be occupational therapists who were currently or had previously used circus in their practice.

Semistructured interviews, guided by preidentified open-ended questions informed by a review of the literature including the historical evolution of circus, were used to provide a space for the interviewees to describe, explain, and explore their practice using circus within their occupational therapy role. The interview guide incorporated H.J. Rubin and I. Rubin's [25] method of asking main questions to ensure the research topic was being addressed and follow-up questions included to elicit more detail with probes used for increased depth and clarification. Interview duration ranged from 45 min to 1.5 h and was conducted and recorded via Zoom. Interviews were transcribed, and accuracy was verified by each participant through member checking. The semistructured interview guide is presented in Table 1.

Ethics approval was obtained from La Trobe University Ethics Committee (HREC S17-031). Pseudonyms were assigned to each participant, and any identifying information conveyed during interviews was removed from transcripts.

The initial data collected between October 2017 and January 2018 were analyzed inductively using interpretive/thematic analysis [24] complementing Stake's [22] case study design continually and cyclically throughout the research process. A second round of data collection was sought approximately 2 years later in November 2019 after the preliminary analysis of the data. The case study participants were asked via email to review their interpreted transcript for accuracy, answer specific probing questions to elaborate on some of their previous responses, and respond to more general follow-up questions that sought to understand any changes in the past years. Braun and Clarke [24] informed the analysis process as becoming familiar with the data through careful reading, generating initial codes, organizing codes into preliminary themes, reviewing themes, defining themes, and then writing up the findings. Individual case transcripts were read multiple times by the research team before cross-case analysis occurred [26]. Investigator triangulation was used to increase methodological rigor and validity. Member checking, review by the research team, and a coding system were applied to promote accuracy. Transparency was achieved by describing in detail the case selection, data collection, and analysis process. The analysis of individual cases revealed both semantic and latent themes reflecting surface-level meanings and more elaborate ideas, assumptions, and interpretations, respectively [24]. The third reading of each case generated initial codes based on content that appeared significant and meaningful or repetitive for each case. This was identified by not only what was said but also the level of depth in which the participant discussed a concept and how often a particular concept was mentioned.

After the initial codes were generated, they were categorized into subthemes representing the connection between individual codes. For example, the *culture* subtheme was assigned to inclusivity, tolerance, and protecting tradition codes. The *challenge* subtheme was assigned to codes of limited funding, lack of research, and colleague buy-in. The *circus skills* subtheme was assigned to group juggling, trapeze, and clowning codes. Thematic analysis included cyclical and multiple processes of cross-checking subthemes. The reflective approach used was informed by complementary aspects of Ayres et al. [23], Leadly et al. [27], and Stanley [28], resulting in the individual and community-focused cases being identified as the most significant overarching themes.

## 3. Findings

Eight occupational therapists from various countries participated, including Australia (*n* = 3), Costa Rica (*n* = 1), the Netherlands (*n* = 1), and the United States of America (*n* = 3). Five out of the eight therapists had previous experience as circus performers before becoming occupational therapists. Those who had not had prior circus experience participated in self-directed professional development to gain knowledge and skills of how to use circus within their practices. The occupational therapists interviewed used circus in similar and different ways for individual and community health and well-being purposes. All case study participants described and referenced theories and models that informed their practice. Participant information is presented in Table 2, detailing participant location, practice setting, and client population.

### 3.1. Identification of Subthemes Across Cases

Eight subthemes were identified across participants' narratives: belonging, joy, skill development, participation, activities, challenges, intentions, and influences. A selection of narrative samples is presented below with identified subthemes bolded in brackets to demonstrate how they are defined within the context of specific examples.

Beatrice described her practice as focusing on addressing the underlying occupational performance issues of children (skill development), including attention, coordination, motor planning, sequencing, problem-solving, and executive function. Vivienne explained that the lack of access to inclusive leisure occupations was a factor in introducing circus as part of her occupational therapy sessions (belonging, intentions). Vivienne stated that a lack of knowledge, exposure to, and evidence about the benefits of circus was an obstacle impeding the use of circus as a therapeutic medium (challenges).

Emily had a circus background before becoming an occupational therapist, which informed her use of circus in her practice (influences). When asked about being able to study circus within her university coursework, she replied, “with a lot of encouragement from me and skepticism from them [professors]” (challenges). Stephanie has personal experience as a circus artist and had been able to collaborate with other circus artists to provide services to her clients (influences). Stephanie used circus activities to improve participation and engagement in various occupations through improving self-esteem, communication, and social skills to create positive change within her adult clients' mental health (participation, skill development).

Henry stated that his practice was influenced by a nonacademic source published by Cirque du Soleil [29]. The social circus model developed by Cirque du Soleil centered around preparing the social agent to facilitate social circus practices (influences). Julie explained her perspectives on the intersection of circus and culture as “I think the culture is, it's kind of a fun, kind of upbeat, spirited … creative group of people that are unique with gifts and talents” (belonging). She believes that the creative and unique aspects of circus promote engagement in occupational therapy, stating, “they are engaged. And they want to do it again, and as soon as they return to the next session, they ask about it.” Julie's priority was for her clients to have fun (participation, joy).

Margaret explained that circus can effectively get children to work on occupational therapy goals by embedding them in enjoyable activities (skill development, joy). “I feel like we are trying to trick these kids into working really hard and not let them know … [or] feel like they are working.” Sarah believed that her personal experience in circus arts enabled her to incorporate circus into her occupational therapy sessions (influences). “I don't know if I would have thought of circus if I hadn't been a circus person first. I knew what it did for me and how powerful it was.”

Cross-case analysis revealed the eight subthemes as similarities between the case study participant narratives. The two overarching themes identified as individual-focused and community health-focused cases revealed the differences between the practice of the participants. The first case, titled “individual-focused circus,” describes the context of participants' use of circus to benefit individuals in their everyday occupations. The second case, titled “community-focused circus,” relates to participants' use of circus to develop community cohesion. The second case references participants' use of circus to influence the broader sociopolitical context or to critique oppressive societal constructs. Seven of eight participants identified individual-focused circus-themed occupational therapy as their primary practice.

### 3.2. Overarching Themes: Circus Cases

#### 3.2.1. Case 1: Individual-Focused Circus

Individual-focused circus facilitates self-confidence, self-esteem, communication, motor skills, and cognitive function, emphasizing the skills an individual needs to participate in self-care, leisure, and productivity occupations. Individual-focused circus is aimed at enabling the person engaging in occupational therapy to gain, maintain, or increase skills to benefit them in a range of meaningful occupations. The similarities in practice among the case study participants using circus for individual-focused outcomes included the different types of circus activities and occupational participation focus areas. Circus activities are composed of distinct “disciplines” such as *aerials* (e.g., silks, trapeze, lyra), *object manipulation* (e.g., juggling, plate spinning, hula hooping, diabolo), *equilibristics* (balancing activities such as tightwire, rolla bolla, and stilt walking), *acrobatics* (similar to floor skills seen in gymnastics), and *clowning* [11]. The description of these activities indicates that circus use as a health intervention promotes visuoperceptual and visuomotor capacities, gross and fine motor control, postural control, balance, and core and limb strength [11].

The circus disciplines used by participants were clowning, object manipulation, balance, and aerial activities. All eight participants described using juggling or some other form of object manipulation in their practice. Five participants (Vivienne, Stephanie, Julie, Margaret, and Sarah) used aerial apparatuses, such as trapeze or aerial silk. Aerial activities were included by therapists who had previous circus experience with these activities. Due to the specific physical environment needed for aerial activities, some therapists made connections with circus schools to enable a conducive environment for aerial experiences. Four participants (Beatrice, Emily, Julie, and Sarah) used balance-based activities such as rolla bolla, stilt walking, or unicycle. Sarah and Henry used clowning activities and games to engage their clients in individual-focused circus-themed occupational therapy. Juggling and aerial were the most frequently used circus disciplines reported by these occupational therapists.

All participants discussed psychosocial development as an occupational performance focus area addressed within their individual-focused sessions. Self-confidence, self-esteem, communication, social skills, and teamwork were terms used relating to psychosocial development. Six participants (Beatrice, Vivienne, Emily, Stephanie, Henry, and Sarah) stated that the ability to participate in noncircus occupations of choice was their therapeutic goal for circus-informed practice, demonstrating occupation-based practice. Beatrice, Vivienne, Stephanie, Julie, Margaret, and Sarah described using circus with individuals to promote motor skills, while Beatrice, Vivienne, Emily, Margaret, and Sarah discussed their use of circus to increase the specific cognitive functions of memory, planning, sequencing, and emotional regulation. Psychosocial development, transferable social and emotional skills, and the circus disciplines of juggling and aerial were the most common features that characterized all case study participants' individual-focused circus therapy sessions.

#### 3.2.2. Case 2: Community Health–Focused Circus

Secondary to the individual-focused case, common threads across participant narratives reflected a community focus. These aspects included culture, acceptance, marginalization, inclusivity, accessibility, meaningful occupation, interpersonal skills, equity, and empowerment. Although meaningful occupation and interpersonal skills also featured strongly in the individual-focused case, within the community-focused case, these terms signified something greater than an individual's performance capacity or ability. The case title, “*community health–focused circus*” reflects participants' reference to community health as something derived from collective or shared individual experiences that reflect something greater than an individual with the potential to impact community relations and influence broader social policies. All eight participants described using circus to contribute to community well-being.

Participants discussed the perceived sociopolitical impacts of their circus sessions as relating to equity, access, the occupational rights of underrepresented communities, and innovative practice. These impacts were recognized in interview conversations relating to participants' understandings of circus culture. A collective understanding of circus as a culture influenced and motivated case study participants' occupational therapy practices. The commonalities expressed specifically related to circus culture inclusivity and change, linking the impacts they believe circus and occupational therapy have within a social–political context. This was indicated in the views shared by Beatrice, who emphasized collaboration, community health, celebration, and acceptance as characteristic features of circus culture.

It is a community, and it's about people coming together for a common cause to celebrate … celebrating individuals and the collective at the same time. I think this is also about acceptance of where you are, and there are no expectations of having to be something.

Sarah felt that in consideration of community well-being, collaboration with a social worker made community-focused interventions more comprehensive. She stated, “I focus on continual adaptation … the social worker that I've partnered with … she focuses more on community development … it's nice to have that partnership with her … I have my occupational therapy lens [and] she has her social work lens.”

All participants believed that through using circus in their occupational therapy practice, the service recipients might embody the characteristics of trust, positive risk-taking, empathy, problem-solving, and teamwork, with experiences of having to rely on and support others, as part of participation and engagement in the variety of circus activities. Similarities existed among all case study participants relating to their use of various circus activities. Circus was viewed through an occupational therapy lens and guided by practice models to create positive changes in individual capacities that were assumed to also impact community well-being.

## 4. Discussion

The findings reveal that the actual practice and aspirations of using circus in their practice were complex for many occupational therapists in this study. The findings reveal a spectrum of circus use where, at one end, the individual-focused case was very tangible, easy to describe, and supported by practice models. In the middle of the spectrum, there was some abstraction in the community health–focused case where participants would report health benefits to the community as intended, implied, or later identified as a secondary outcome not explicitly intended or measured. Further along the community health–focused spectrum, group-based circus sessions with explicit local community health goals were evident. At the far end of the spectrum, more implied than described, was a sociopolitical intent. The purpose of this study was to increase understanding of how circus is being used by occupational therapists. A significant finding was an emphasis on individuals, albeit a greater community focus existed. How the benefits of using circus within occupational therapy practice can be emphasized in collective community occupations was not evident.

**Figure 1:**
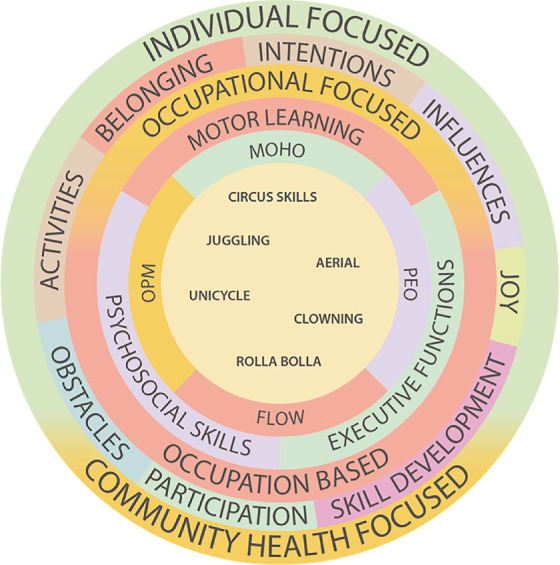
Practice trends among case study participants.

Figure 1 represents the components of circus use described across all case study participants. The outermost ring features two overarching themes: the individual-focused and community health–focused circus cases, inclusive of all aspects of the rings within. The imbalance of shadings within this ring conveys circus use as being more individually focused than community health focused. The second ring identifies the subthemes that emerged in the final stages of analysis. The third ring represents the tension between occupation-based and occupation-focused practice, where two significant trends emerged. All the case study participants provided examples of intending to use circus for performance capacity development and participation simultaneously, demonstrating a complementary use of circus in both occupation-based and occupation-focused ways.

The presence of significantly more examples and descriptions of circus being used to promote individual capacities was evident. Motor learning, psychosocial skills, and executive functions represent the performance capacities most often addressed by participants. The next ring features the models influencing participants' practices and evidence in the cross-case analysis. The innermost circle represents the circus activities used by case study participants.

Figure 1 can be summarized as a depiction of circus skills being facilitated through the lens of occupational therapy practice models to address specific performance capacities. The therapists' mixed occupation-based and occupation-focused intentions contribute to improving the health of individuals and communities as clients. The outermost ring demonstrates the predominant use of circus for individual-focused health.

How occupational therapy models inform the practice of occupational therapists using circus was a significant finding of this study, for it highlighted the potential differences between occupational therapy–informed circus and social circus. The occupational therapy models and frames of reference outlined below informed case study participants' professional reasoning when responding to specific occupational participation and performance issues impacting individual and community well-being. The widely used and primarily Western-developed occupational therapy theoretical models include the model of human occupation (MOHO) [30], the person–environment–occupation model (PEO) [31], and the occupational performance model (OPM) [32]. The MOHO [30] considers the individual, their occupations, and how these relate to their environment, including consideration of motivations, roles, habits, and personal performance capacities. The PEO [31] emphasizes that occupational performance is shaped by the interaction between person, environment, and occupation. The OPM [32] assumes that occupational engagement gives individuals a sense of competence, autonomy, and meaning of existence. Although these three occupational therapy models can be applied in macrolevel practice, the participants in this study predominantly referred to them in context of individual-focused, remediation, and performance capacity–oriented practices.

Participants' remediation focus reflects the profession's familiarity and comfort with using individual-based independence-focused practice models. The components of Figure 1 are significant for two reasons. Firstly, the components can be used in future studies to measure the effectiveness of individual-focused circus practices on motor learning, psychosocial skills, and executive functions. Secondly, they highlight the gaps and potential for more community-centered and occupational-focused practice using circus.

There is significant potential for circus use by occupational therapists to bridge the gap between individual-focused and community-focused practice. Although all case study participants alluded to an awareness of community-centered influences inherent within their individual-focused practices, Henry was an exception, giving equal weight to the community-focused and individual-focused intentions of his practice. Henry's circus sessions promoting development of social skills demonstrated how teaching these skills could lead to improvement in the way communities and the people within them coexist. “These activities help you to communicate better with others, to open your mind to other possibilities, not just the ones you have.” Henry spoke of both traditional occupational therapy models, such as the MOHO, and social circus literature informing his circus-themed occupational therapy practices.

While not mentioned by participants, the CanMOP [1] may be particularly relevant as a guide for community-centered circus use. Participants' focus on their clients' participation over skill mastery during engagement in various circus skills aligns with more recent conceptualizations of participation conveyed in the CanMOP [1], such as cultural relevance and community development. A focus on cultural relevance and community development demonstrates a shared understanding among the occupational therapists in this study of participation going beyond occupational performance capacities.

When considering community health, occupational justice is often relevant. Occupational justice includes environmental, social, and health equity. It is a societal need that extends beyond individual and family-focused services when providing occupational therapy services for organizations, communities, and populations [21, 33]. Having occupational therapists working in community health contexts is beneficial for raising awareness relating to the impacts of occupational therapy, influencing policy, and responding to the social and health problems that communities experience [21]. Capitalist structures [5, 34, 35], exploitation of human and nonhuman beings [5, 34], empowerment through occupational choice and autonomy [3, 6, 35, 36], hegemonic social and family systems [5, 6], socially and culturally diverse, while simultaneously segregated by class, race, occupation, and gender [5, 35], are all part of the complex and multifaceted history of circus that included social transformations, progressions, and injustices [5].

Collaboration among occupational therapists and social circus advocates has the potential to advance evidence-based practice in social circus and community development targeting occupational justice. There are potential positive implications for individuals and communities in a global context with increased understanding and exploration of the collaboration of marginalized individuals, circus communities, and health professions, including occupational therapy. Collaboration and a prohumanity response to sociopolitical events have been the continual call to action from thought leaders in occupational therapy [1, 2, 37], as well as characterized in the history of circus arts as more than entertainment but also a form of societal health promotion. Collaboration is an asset needed to cocreate infrastructure for a healthy environment that is less susceptible to the impending and potentially dangerous social and political environmental forces.

Beneficiaries of a social circus program in Ecuador [38] reported the value of participation and engagement in the program, which enabled them to transform how they relate to others and form their future. One participant from this social circus program commented on the impact the program had on them wanting to interact with society. They stated, “when you recognize yourself as an individual, then you can be part of society and have some sort of impact. Change starts with oneself” ([38], p. 916). Individual and community are interdependent, which lends justification to the vital role of occupational therapy in addressing individual performance capacities and community-focused practice initiatives.

## 5. Study Limitations and Strategies to Enhance Trustworthiness

As an occupational therapist who uses circus in her own individual and community-focused practice, the first author acknowledges her bias. Processes to maintain reflexivity throughout data collection were adopted to mitigate the personal bias of the first author and included journaling and challenging assumptions and beliefs. Interviews were the only form of data collection, limiting the variety of data sources used to answer the research question. There may have been methodological bias in asking interviewees more questions about the challenges that impacted their practice than what solutions and recommendations they had for increasing circus use in occupational therapy.

## 6. Recommendations

The findings suggest that further research into the effectiveness and evaluation of the therapeutic benefits of circus use by occupational therapists is required. Participants in this study used circus as a therapeutic medium to increase individual capacities relating to social, emotional, physical, and cognitive health. The impact of circus interventions on service recipients' capacities and transference to other individual and collective community occupations remains unclear and warrants further research.

There is a need for occupational therapists to collaborate with other disciplines working to address occupational injustice and promote occupational rights. Greater collaboration between social activists, circus practitioners, and occupational therapists will enable better outcomes in addressing occupational injustice and promoting occupational rights.

## 7. Conclusion

The findings of this study suggest that desire and intention are present among occupational therapists to pursue a broader community and occupational justice–focused agenda specifically using circus considering the focus of this paper, but this may be obscured by therapists' professional experience being grounded in working with individuals and groups to achieve individual performance goals. Because it is still an emerging area of practice, how many therapists are using circus and in what ways are still largely unknown. More research is needed to further explore circus use by occupational therapists in community development contexts and the potential impacts on occupational rights.

## Figures and Tables

**Table 1:** Semistructured interview guide.

1. Tell me a bit about yourself and your career as an occupational therapist.
2. Tell me about how you use circus in your practice as an occupational therapist.
3. What motivated you to use circus in your practice?
4. Can you describe how you address specific occupational issues using circus as an intervention?
• Within individual treatment contexts
• Within community-based and/or focused (centered) contexts
• Within sociopolitical contexts
5. What factors inhibit circus being utilized as an occupational therapy intervention?
6. Do any models or theories relate well to circus in occupational therapy or underpin your practice and why?

**Table 2:** Participant characteristics.

**Participant pseudonym**	**Country**	**Environment or settings**	**Populations receiving occupational therapy**
Beatrice	Australia	Primary and secondary school/sensory classroom	Children/adolescents
Vivienne	Australia	Primary and secondary school/circus school	Children/adolescents/autism
Emily	Netherlands	Circus school/orphanages	Children/adolescents
Stephanie	Australia	Community mental health center/circus schools	Adults/mental health
Henry	Costa Rica	Community center	Children/adult women
Julie	United States	Primary and secondary school/private practice clinic	Children
Margaret	United States	Primary and secondary school/private practice clinic	Children/adolescents
Sarah	United States	Primary and secondary school/community center/school for deaf and hard of hearing/university/academic conferences	Children/adolescents/adults/women cancer survivors/intellectual disability/traumatic brain injury/heard of hearing/mental health

## Data Availability

The authors declare that the data that support the findings of this study are available on request from the corresponding author. The data are not publicly available due to privacy or ethical restrictions.
